# A Holling Functional Response Model for Mapping QTLs Governing Interspecific Interactions

**DOI:** 10.3389/fgene.2021.766372

**Published:** 2021-10-15

**Authors:** Xiao-Yu Zhang, Huiying Gong, Qing Fang, Xuli Zhu, Libo Jiang, Rongling Wu

**Affiliations:** ^1^ College of Science, Beijing Forestry University, Beijing, China; ^2^ Faculty of Science, Yamagata University, Yamagata, Japan; ^3^ Beijing Advanced Innovation Center for Tree Breeding by Molecular Design, Center for Computational Biology, College of Biological Sciences and Technology, Beijing Forestry University, Beijing, China; ^4^ Center for Statistical Genetics, The Pennsylvania State University, Hershey, PA, United States

**Keywords:** interspecific interaction, quantitative trait loci, differential equation, microbial growth, system mapping

## Abstract

Genes play an important role in community ecology and evolution, but how to identify the genes that affect community dynamics at the whole genome level is very challenging. Here, we develop a Holling type II functional response model for mapping quantitative trait loci (QTLs) that govern interspecific interactions. The model, integrated with generalized Lotka-Volterra differential dynamic equations, shows a better capacity to reveal the dynamic complexity of inter-species interactions than classic competition models. By applying the new model to a published mapping data from a competition experiment of two microbial species, we identify a set of previously uncharacterized QTLs that are specifically responsible for microbial cooperation and competition. The model can not only characterize how these QTLs affect microbial interactions, but also address how change in ecological interactions activates the genetic effects of the QTLs. This model provides a quantitative means of predicting the genetic architecture that shapes the dynamic behavior of ecological communities.

## Introduction

Understanding the internal workings of ecological communities is of fundamental importance to predict community dynamics and improve ecosystem services (Whitham et al., 2006; Vellend, 2020). Mounting evidence shows that genes play a pivotal role in shaping community structure, organization, and function (Whitham et al., 2006; Bailey et al., 2009; Hersch-Green et al., 2011; Miner et al., 2012; Crutsinger, 2016; Salazar et al., 2019; Wimp et al., 2019). For example, in response to predator-borne kairomones, some genes in Daphnia are activated, showing a higher-level expression than usually (Schwarzenberger et al., 2009; Miyakawa et al., 2010). To systematically characterize specific community genes, their number, chromosomal locations, and effect size, a powerful mapping approach has emerged through designing a community-ecological experiment using a mapping population (Jiang et al., 2018; Jiang et al., 2020).

Genetic mapping is a statistical approach widely used to map and identify genes, known as quantitative trait loci (QTLs), which control complex traits (Flint et al., 2005; Atwell et al., 2010; Consortium et al., 2015; Zeng et al., 2018). By integrating the mathematical aspect of trait formation, functional mapping has been developed to reveal the spatiotemporal pattern of the genetic architecture underlying phenotypic variation and evolution (Ma et al., 2002; Wu et al., 2005; Wu and Lin, 2006; Zhao et al., 2012; Li and Sillanpaa, 2015). The interpretable advantage of functional mapping has been leveraged to capture the biological rule governing how the components constituting a complex trait are interconnected, interdepended and interacted to mediate trait variation. A so-called system mapping approach has been assembled to map the genetic machineries underlying such component-component interconnections (Gai et al., 2011; Bo et al., 2014; Sun and Wu, 2015).

More recently, Jiang et al. (2018) integrated systems mapping and evolutionary game theory into a unified model to study the genetic control of species-species interactions (including cooperation and competition) in ecological communities. This cooperation-competition mapping (CoCoM) model differs from conventional systems mapping, in that the former needs to model genetic effects from two or multiple interacting species, whereas the latter only needs to consider one single genome of the species studied. By introducing the notion of evolutionarily stable strategy proposed by Smith and Price, CoCoM incorporates a system of nonlinear Lotka-Volterra (nLV) predator-prey equations (May, 1975) to partition the phenotypic value of each species in communities into its independent and dependent components. The independent component is one that occurs when this species is assumed to be in isolation, and the dependent component reflects the effect due to the influences of other species on this species. Thus, CoCoM is equipped with a capacity to reveal how much these two components contribute to phenotypic variation and how their relative contributions are controlled reciprocally by the genes of a pair of species. However, as the first model of its kind, CoCoM uses a simple from of nLV equations that may not adequately capture the chaotic complexity of ecological communities (May, 1975; Cushing, 1980; Arneodo et al., 1982; Panetta, 1996; Huisman and Weissing, 1999; D’Onofrio, 2002; Zeng et al., 2005).

The motivation of this study is to expand the utility of CoCo to a broader context that is allowed to be periodically oscillated. To model the perturbation of the community, we introduce a Holling-type functional response model to characterize impulsive perturbations of the nLV system. The Holling-type model has been widely used to study community behavior and dynamics at different scales (Liu and Chen, 2003; Zhang et al., 2005; Leeuwen et al., 2007). By analyzing a published dataset from an ecological experiment including monocultures and co-cultures of two bacterial species, the new model identifies previously unidentified genetic loci for microbial cooperation and competition. Comparing the difference of genetic architecture in socially isolated monocultures and socialized co-cultures gains new insight into the genetic mechanisms underlying microbial interactions. Computer simulation has been performed to validate the statistical properties of the new model.

## Materials and Methods

### Mapping Materials

We used a published experimental data (Jiang et al., 2018) to validate the utility of our model. The experiment, conducted with 45 strains from each of two bacterial species, *Escherichia coli* and *Staphylococcus aureus*, includes 45 strain-specific monocultures of each species and 45 interspecific co-cultures using independent strain pairs of two species. The abundance of each strain in monoculture and co-culture was measured repeatedly at 16 time points during growth process.

### Holling Equations

Trait formation is a biological process that includes the increase of trait value with time, i.e., growth. Based on the biological rule governing growth, several growth equations, including logistic, Richards, and Gompertz, have been proposed to quantify the pattern of growth (Zwietering et al., 1990; West et al., 2001; Palacios et al., 2014). Functional mapping incorporates the growth equation to map QTLs for trait formation and development, showing increasing biological relevance and statistical power (Ma et al., 2002; Wu et al., 2005; Wu and Lin, 2006; Zhao et al., 2012; Li and Sillanpaa, 2015). However, these growth equations describe the growth of an organism without considering its biological surrounding where the other organisms would exert effects on its growth. By treating interactive organisms as a complex system, several nLV-based ordinary differential equations (ODEs) have been developed to model how different species interact with each other to determine the system (Fujikawa et al., 2014). nLV equations, widely applied to study prey-predator relationships in ecological communities (Hernández-Bermejo and Fairén, 1997; Terborgh and Estes, 2010), have been implemented to map cooperation and competition QTLs (Jiang et al., 2018; Jiang et al., 2020).

Beginning in the late 1950s, Holling conducted an experiment to investigate how a predator’s rate of prey capture is related to prey density (Holling, 1959a; Holling, 1959b). In the resulting series of seminal articles, Holling identified three general categories of functional response: Types I, II, and III (Holling, 1965; Morozov, 2010). Type I is the simplest: capture rate increases in direct proportion to prey density until it abruptly saturates. Type II is similar in that the rate of capture increases with increasing prey density, but in contrast to the linear increase of Type I, Type II approaches saturation gradually. Type III is similar to Type II except at a low prey density, where the rate of prey capture accelerates. Many experiments have proved that Holling-type functional response plays a key role in understanding predator-prey relationships (Ji et al., 2009; Tewa et al., 2013) and can fill the gap of nLV equations. Most studies view type II functional response as the basis for choosing an optimal foraging model because it is more likely to occur in communities where population dynamics is driven by predation (Ji et al., 2009).

Here, we integrate Holling type II functional response and nLV-based ODEs (HollinLV) to model the interaction and coordination mechanisms of two populations in communities. Assume that A and B are two species used to conduct an ecological experiment in which each species is grown in socially isolated monocultures and, also, both are grown in a socialized co-culture. Let E and S be the abundance of species A and species B, respectively, in a community. The HollinLV model is expressed as follows:

$$\{\begin{array}{c} \frac{dE}{dt}=r_{e}E\left(1-\frac{E}{K_{e}}\right)+r_{e}E\left(\frac{\alpha_{E\leftarrow S}}{1+E}\right)S\\ \frac{dS}{dt}=r_{s}S\left(1-\frac{S}{K_{s}}\right)+r_{s}S\left(\frac{\alpha_{S\leftarrow E}}{1+S}\right)E \end{array}$$ (1)

where r_e_ and r_s_ represent the Malthusian growth rates of species A and species B, respectively; K_e_ and K_s_ are an intrinsic-carrying capacity of two different species; $\;\alpha_{E\leftarrow S}$ , $\;and\;\alpha_{S\leftarrow E}$ represent the scalar parameter values that describe how one species affects the other through competition or cooperation in the co-culture. According to Eq. 1, the growth trajectories of each species is the summation of two parts:

$$\{\begin{array}{c} \frac{dE}{dt}=r_{e}E\left(1-\frac{E}{K_{e}}\right)\\ \frac{dS}{dt}=r_{s}S\left(1-\frac{S}{K_{s}}\right) \end{array}$$ (2A)

and

$$\{\begin{array}{c} \frac{dE}{dt}=r_{e}E\left(\frac{\alpha_{E\leftarrow S}}{1+E}\right)S\\ \frac{dS}{dt}=r_{s}S\left(\frac{\alpha_{S\leftarrow E}}{1+S}\right)E \end{array}$$ (2B)

Following Jiang et al. (2018), we define the first part as the *independent* growth of a species that occurs when this specie is assumed to grow in isolation, which corresponds to the growth of this species in monoculture, and the second part as the dependent growth of a species, determined by the interaction of its co-existing species through a certain mechanism. The degree of this dependence is described by interaction scalar parameters $\alpha_{E\leftarrow S}\;\mathrm{and}\;\alpha_{S\leftarrow E}$ .

According to population ecology theory, there are mainly three types of interaction among species (Svenning et al., 2014): 1) neutral interaction, i.e., there is no interaction between species and they are independent of each other; 2) positive interaction, including commensalism and mutualism. If only one side is favorable and there is no influence on the other side, it is called partial benefit symbiosis; if both sides are favorable, it is called mutualism; and 3) negative interaction, including antagonism, predation and amensalism. Antagonism is opposite to mutualism; predator preys on other species, which is the traditional relationship between preying and being preyed on; amensalism means that the existence of one species has an inhibitory effect on the other, but the second no effect on the first.

By estimating the interaction and scale parameters, we can quantify the degree of each possible interaction. While the interaction parameters determine whether and in which direction the dependent growth occurs, scale parameters, $\alpha_{E\leftarrow S}\;\mathrm{and}\;\alpha_{S\leftarrow E}$ , determine how the interaction strengthens or weakens with growth development. A positive or negative, $\alpha_{E\leftarrow S}\mathrm{or}\alpha_{S\leftarrow E}$ value indicates that this species is benefitted or harmed by another species. If the value of $\alpha_{E\leftarrow S}\;\mathrm{or}\;\alpha_{S\leftarrow E}$ is zero, this means that the interaction does not depend on another species. If they are also zero, which means that the two species are not affected by one another.

### A Mapping Framework

In the co-culture environment, both HollinLV and CoCoM models consider the interactions between species. Here, we incorporate HollinLV into systems mapping, an approach of mapping complex traits by treating complex traits as a dynamic system (Gai et al., 2011; Bo et al., 2015; Sun and Wu, 2015). Consider two mapping populations each from a different species, A or B. Each population has n genome-wide genotyped members, grown individually in monoculture and in co-culture in interspecific pairs. Let $E_{i}=\left(E_{i}\left(1\right),\cdots ,E\left(T\right)\right)$ and $S_{i}=\left(S_{i}\left(1\right),\cdots ,S_{i}\left(T\right)\right)\left(i=1,\cdots ,n\right)$ denote the abundance of n pairs from species A and B measured at time T in co-culture. Consider a genetic locus with two genotypes A and a for species A and two genotypes B and b for species B. Pairing species A and B generates four interspecific genotype combinations AB, Ab, aB, and ab, with observations denoted as n_AB_, n_Ab_, n_aB_, and n_ab_, respectively. We formulate the likelihood function for the abundance data of interspecific pairs at the locus as

$$L=\overset{n_{AB}}{\underset{i=1}{\prod }}f_{AB\;}\left(E_{i},S_{i};\theta_{AB}\right)\times \overset{n_{Ab}}{\underset{i=1}{\prod }}f_{Ab\;}\left(E_{i},S_{i};\theta_{Ab}\right)\times \overset{n_{aB}}{\underset{i=1}{\prod }}f_{aB\;}\left(E_{i},S_{i};\theta_{aB}\right)\times \overset{n_{ab}}{\underset{i=1}{\prod }}f_{ab\;}\left(E_{i},S_{i};\theta_{ab}\right)$$ (3)

where θ is the HollinLV parameter ( $r_{e},K_{e},\;\alpha_{E\leftarrow S},\;r_{s},\;K_{s},\;\alpha_{S\leftarrow E}$ ) describing abundance, $f_{j}\left(E_{i},S_{i};\theta_{j}\right),j\in \left(AB,Ab,ab,aB\right)$ is a bivariate longitudinal normal distribution specified by genotype-specific mean vectors,

$$\mu_{j}=\left(\mu_{j1};\mu_{j2}\right)=\left(\mu_{j1}\left(1\right),\cdots ,\mu_{j1}\left(T\right);\mu_{j2}\left(1\right),\cdots ,\mu_{j2}\left(T\right)\right)$$ (4)

and covariance matrix,

$$\text{Σ}=\left(\begin{array}{cc} {\text{Σ}}_{11} & {\text{Σ}}_{12}\\ {\text{Σ}}_{21} & {\text{Σ}}_{22} \end{array}\right),$$ (5)

with variance matrices of abundance over time on the middle diagonal and covariance matrices of abundance between two species on the non-diagonal.

We model the vector structure of Eq. 4 by HollinLV Eq. 1 and the structure of covariance matrix Eq. 5 by a first-order structured antedependence [SAD(1)] statistical model (Zimmerman and Nunez-Anton, 1997; Zhao et al., 2005a; Zhao et al., 2005b). We implement a hybrid of the Nelder-Mead simplex algorithm and the fourth-order Runge-Kutta algorithm to solve the likelihood Eq. 3. The maximum likelihood estimates (MLEs) of ODE parameters and SAD(1) parameters can be obtained.

### Hypothesis Testing

The existence of significant QTLs associated with interspecific interactions can be tested by a log-likelihood ratio (LR) approach. To do so, we formulate two hypotheses expressed as

$$\begin{array}{l} H_{0}:\;\Theta_{j}\equiv \Theta ,\\ H_{1}:\;\Theta_{j}\neq \Theta , \end{array}$$ (6)

for j∈(AB,Ab,ab,aB) , under which the likelihood values L_0_ and L_1_ are calculated, respectively. The test statistic LR is calculated as

$$\mathrm{LR}=2\left(\log L_{1}-\log L_{0}\right)$$ (7)

which is compared with the critical threshold. Note that the null hypothesis states that these ODE parameters are genotype invariant. The rejection domain of the likelihood ratio statistics is, W={LR≥c} , where c satisfies

$$P_{\theta }\left(\mathrm{LR}\geq c\right)\leq \alpha$$ (8)

When the value of the test statistic LR is in the rejection domain, the null hypothesis is rejected, which suggests that there are differences in the abundance from different interspecific combination genotypes. We can also test whether a significant QTL affects the independent growth by formulating

$$\begin{array}{l} H_{0}:\left(r_{ej},k_{ej},r_{sj},k_{sj}\right)\equiv \left(r_{e},k_{e},r_{s},k_{s}\right)\\ H_{1}:\left(r_{ej},k_{ej},r_{sj},k_{sj}\right)\neq \left(r_{e},k_{e},r_{s},k_{s}\right) \end{array}$$ (9)

or the interaction growth by formulating

$$\begin{array}{l} H_{0}:\left(r_{ej},\alpha_{E\leftarrow Sj};r_{s},\alpha_{S\leftarrow Ej}\right)\;\equiv \left(r_{e},\alpha_{E\leftarrow S},r_{s},\alpha_{S\leftarrow E}\right),\\ H_{1}:\;\left(r_{ej},\alpha_{E\leftarrow Sj};r_{s},\alpha_{S\leftarrow Ej}\right)\neq \left(r_{e},\alpha_{E\leftarrow S},r_{s},\alpha_{S\leftarrow E}\right), \end{array}$$ (10)

The LRs for the above two pairs of hypotheses are calculated and compared with the critical thresholds, respectively.

According to Jiang et al. (2018), we partition the overall genotype values of significant SNP pairs into direct effects, indirect effects, and genome-genome epistatic effects. The direct effect describe how a QTL directly affects the abundance dynamics of each species from its own genome. The indirect effect describes how a QTL indirectly affects the abundance dynamics of each species from the genome of its interactive genome. The epistatic effect characterizes how the interaction of alleles from different species affects the abundance dynamics of each species. Traditional genetic mapping can only estimate and test the indirect genetic effects, whereas our model can estimate and test all these three effects, which thus can gain new insight into the genetic architecture of interspecific interactions in ecological communities.

## Results

### Fitting Growth Curves

We used Gompertz, Logistic and Richards growth equations, Jiang et al. (2018) CoCoM, and our HollinLV to fit the mean growth curves of microbial abundance for 45 strains from each species in co-culture. We compared the performance of these models by calculating AIC (Akaike information criterion), BIC (Schwarz criterion), and HQ (Hannan Quinn criterion) (Table 1). As shown, CoCoM and HollinLV perform better than three classical growth equations, suggesting that it is crucial to consider the impact of interactive species on the growth of a given species in a community. Our HollinLV shows a better performance than CoCoM. In order to show the fitting effects specifically, the actual values and fitting curves of 45 pairs of co-culture samples are plotted, and the goodness of fit ( $R^{2}$ ) are calculated in Supplementary Figures S1, S2, indicating the samples achieve good fitting effects.

**TABLE 1:** The estimated parameters of various growth equations, the goodness of fit $R^{2}$ , adjusted $R^{2}$ , and their evaluation information based on akaike information criterion (AIC), bayesian information criterion (BIC), and hannan-quinn information criterion (HQIC). Gompertz, Logistic, Richards, DDHR, and CoCoM models were used to fit the average abundance of *E. coli* and *S. aureus* under co-culture.

Gompertz		Logistic		Richards	
*E. coli*	*S. aureus*	*E. coli*	*S. aureus*	*E. coli*	*S. aureus*
K = 24.07 ± 2.07	K = 21.66 ± 2.05	K = 23.94 ± 1.96	K = 21.56 ± 1.95	K = 24.74 ± 4.08	K = 21.82 ± 4.05
a = −0.11 ± 0.16	a = −0.22 ± 0.17	a = 0.29 ± 0.21	a = 0.16 ± 0.26	a = 0.90 ± 7.16	a = 0.25 ± 3.73
b = 0.1 ± 0.18	b = 0.18 ± 0.20	b = 0.22 ± 0.26	b = 0.21 ± 0.25	b = 0.10 ± 0.13	b = 0.16 ± 0.09
				m = −1.25 ± 2.71	m = 0.65 ± 4.81
$R^{2}$ = 0.9555		$R^{2}$ = 0.9560		$R^{2}$ = 0.9606		
$adj.R^{2}$ = 0.9485		$adj.R^{2}$ = 0.9490		$adj.R^{2}$ = 0.9518		
AIC = 12.3908		AIC = 12.3671		AIC = 12.2362		
BIC = 12.6317		BIC = 12.6080		BIC = 12.5574		
HQ = 12.3024		HQ = 12.2786		HQ = 12.1183		

CoCoM			DDHR			
*E. coli*	*S. aureus*		*E. coli*		*S. aureus*	
$r_{e}=$ 0.28 ± 0.14	$r_{s}=$ 0.25 ± 0.11		$r_{e}=$ 0.46 ± 0.19		$r_{s}=$ 0.46 ± 0.19	
$K_{e}=$ 83.09 ± 4.10	$K_{s}=$ 62.30 ± 6.59		$K_{e}=$ 22.67 ± 3.56		$K_{s}=$ 20.76 ± 3.62	
$\alpha_{E\leftarrow S}=$ −2.78 ± 0.33	$\alpha_{s\leftarrow E}=-1.73$ ±0.63		$\alpha_{E\leftarrow S}=$ −1.96 ± 0.79		$\alpha_{s\leftarrow E}=-1.96$ ±1.84	
$R^{2}$ = 0.9607			$R^{2}$ = 0.9633			
$adj.R^{2}$ = 0.9545			$adj.R^{2}$ = 0.9575			
AIC = 6.9525			AIC = 6.8855			
BIC = 7.1934			BIC = 7.1264			
HQ = 6.8641			HQ = 6.7971			

**FIGURE 1:**
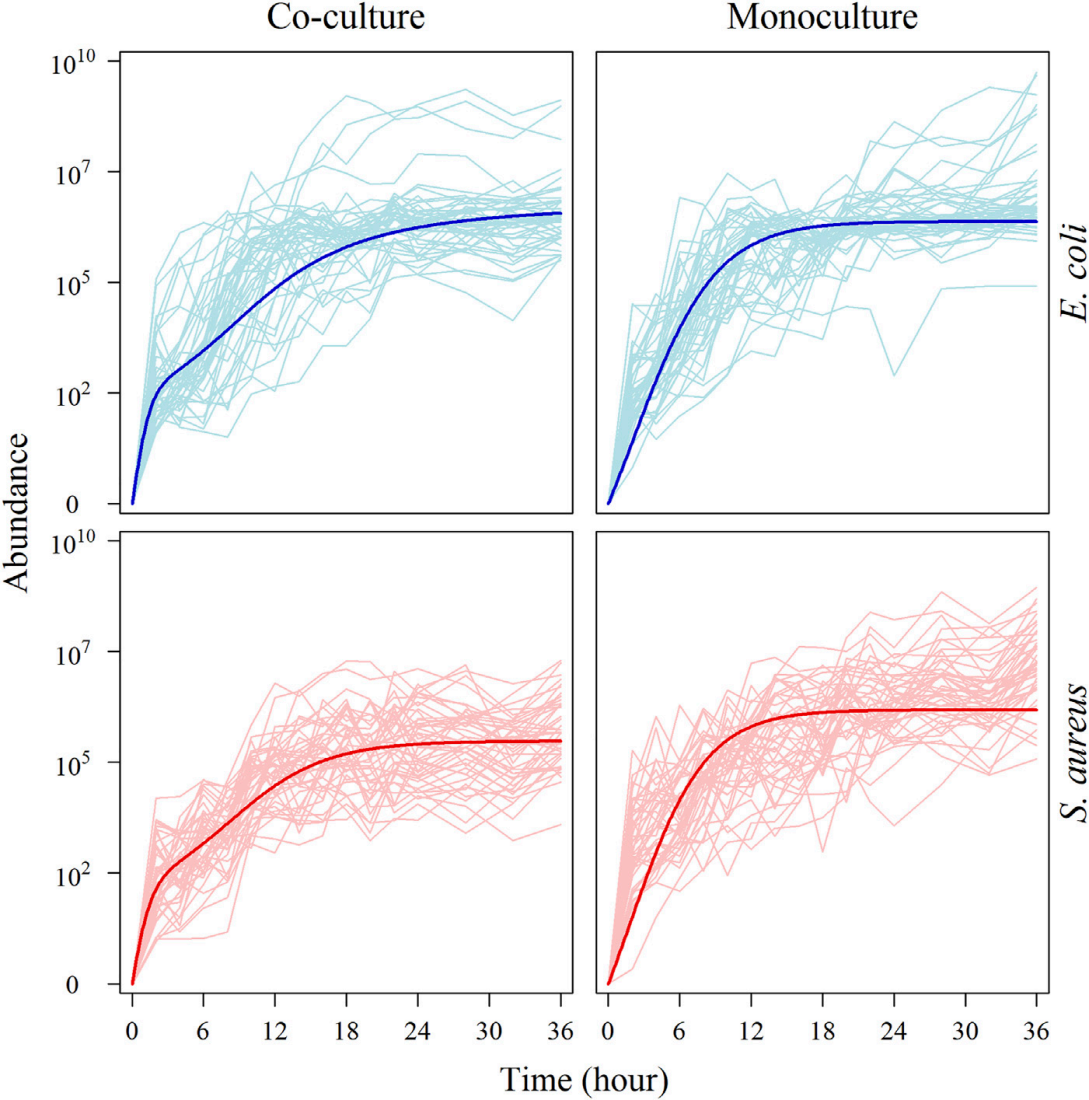
Growth curve of microbial abundance under monoculture and co-culture. Microbial abundance of individual strains from each species was observed in monoculture and co-culture during the first 36 h after culture. The **upper:** the abundance curve of *E. coli* under monoculture and co-culture, respectively. The thick blue line is the average growth curve of *E. coli*. The **lower:** the abundance curve of *S. aureus* under monoculture and co-culture, respectively. The thick red line is the average growth curve of *S. aureus*.

Figure 1 illustrates the abundance trajectories of the same species in co-culture and monoculture. We find that the growth of two species in co-culture is affected by each other to a certain extent. The most significant change of growth trajectories displays in the timing of maximum relative growth rate; microbes from both species tend to enter fast-growing stages in co-culture than in monoculture. However, the degree to which *S. aureus* growth is affected by *E. coli* is greater than that to which *E. coli* growth is affected by *S. aureus*. This suggests that, as compared to *E. coli*, the growth of *S. aureus* is more sensitive to the biotic environment. All these biotically induced changes imply the existence of specific genes that are activated by species coexistence.

### How QTLs Are Activated by Species Coexistence

We apply HollinLV to map QTLs for the microbial abundance of each species in co-culture, in a comparison with those detected in monoculture. To map growth trajectories in co-culture, we implement two ODEs of Eq. 1, each for a different species, into a mapping framework, whereas the corresponding mapping of growth in monoculture is based on the implementation of two ODEs in Eq. 2A.

**FIGURE 2:**
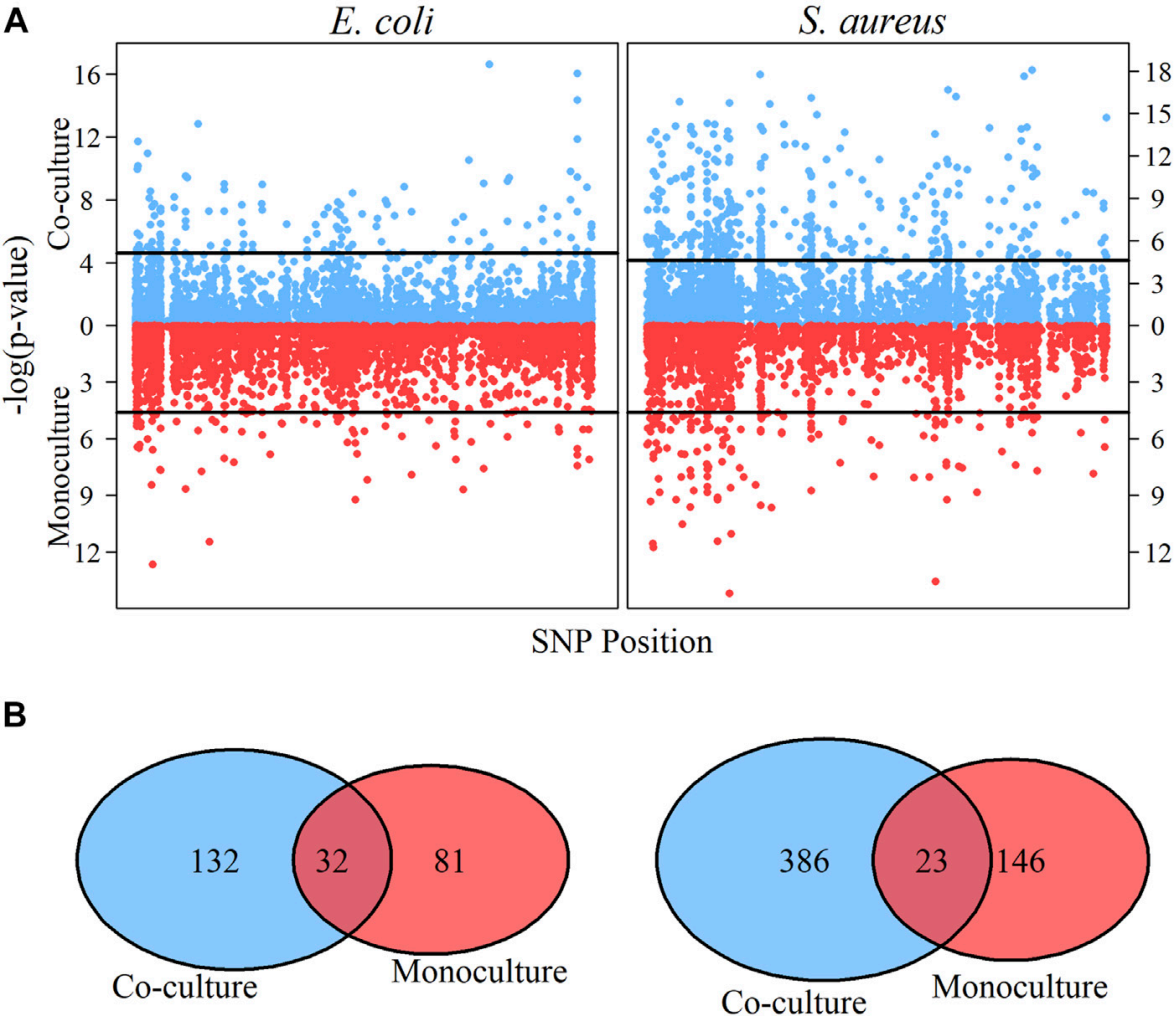
Identification of significant QTLs and comparative analysis for *E. coli* and *S. aureus* under different culture. **(A)** Manhattan plots in co-culture **(the upper part of A)** and monoculture **(the lower part of A)** for *E. coli* and *S. aureus*, respectively. From Manhattan plots A, significant QTLs are detected through significance tests. The horizontal lines are the genome-wide critical threshold at 0.01significance level. **(B)** Comparison of significant QTLs for microbial growth under different culture conditions. The blue color represents co-culture condition and the red color represents monoculture condition.

Figure 2A shows the genomic distribution of QTLs detected for each species in different cultures. We identify more growth QTLs for *S. aureus* than *E. coli* in both monoculture and co-culture, but for both species, a number of new QTLs are activated from monoculture to co-culture (Figure 2B). In monoculture, there are a total of 81 QTLs that influence the growth trajectory of *E. coli*, but this number increases to 132 in co-culture, of which 32 are the common QTLs for both types of cultures. In other words, 49 QTLs are specifically expressed in monoculture, whereas 100 only function in co-culture. In *S. aureus*, 123 QTLs are monoculture-specific, 363 are co-culture-specific, and 23 are common to both cultures.

**FIGURE 3:**
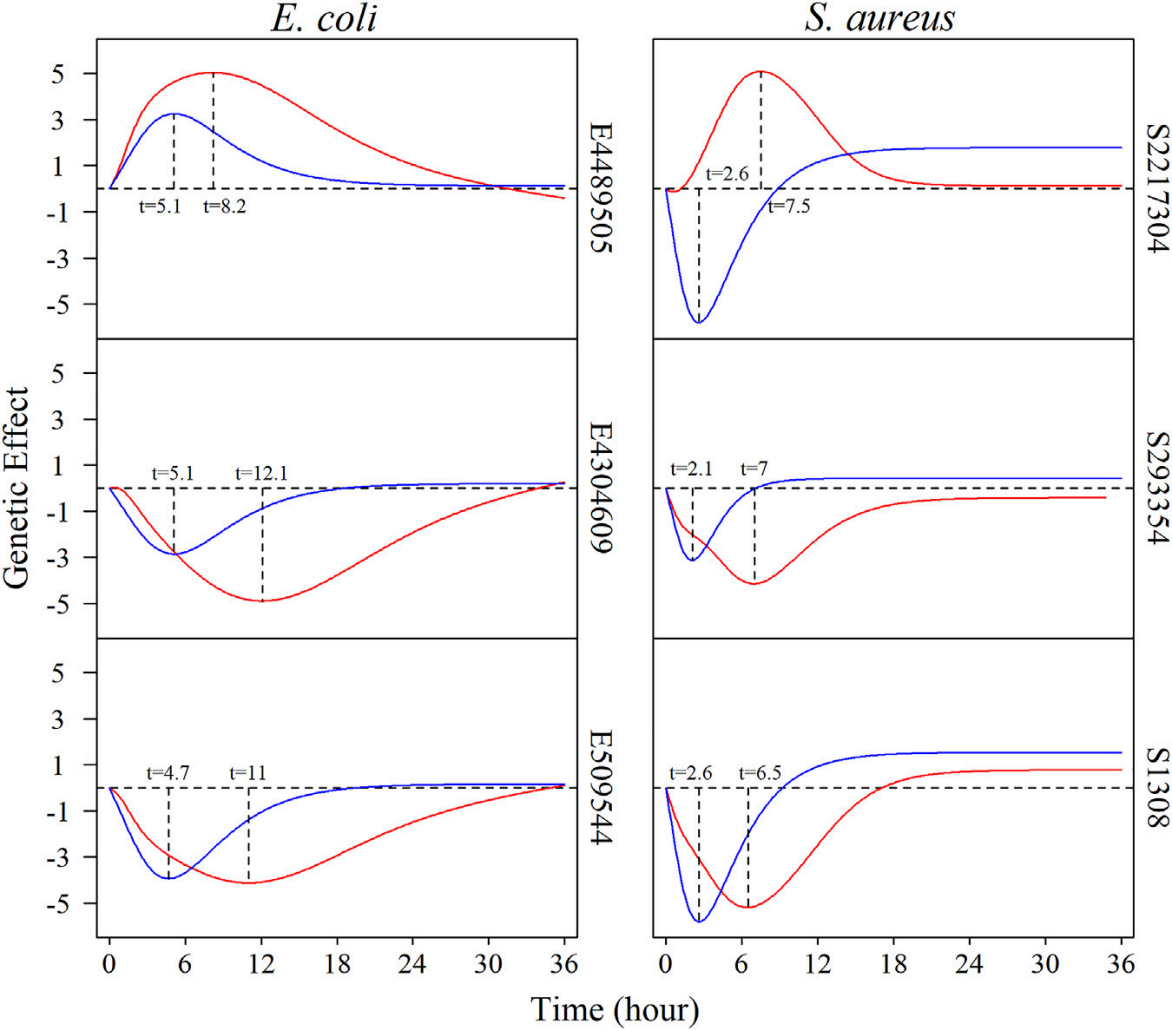
The genetic effect curves regulated by common QTLs which are significant under both single culture and co-culture conditions. The **left:** the effect curves of *E. coli* of E4489505, E4304609, and E509544, respectively. The **right:** the effect curves of *S. aureus* of S2217304, S293354, and S1308, respectively. The red lines represent the genetic effect under co-culture, and the blue lines represent the genetic effect under monoculture.

We estimate the genetic effect curves of each QTL in monoculture and co-culture for both species. Figure 3 depicts how three representative common QTLs from each species change their genetic effects during growth. Overall, the temporal effects of QTLs cyclically change with time; i.e., a QTL increases its effect after culture and then monotonically decrease its effect at a certain time point. For the culture-common QTLs, the pattern of time-varying genetic effects depends on culture type. It is interesting to note that the same QTL displays a peak of its effect earlier in co-culture than in monoculture. For example, QTL E4304609 reaches a maximum effect when *E. coli* was cultured for 5 h in co-culture, but the timing of its maximum effect occurs at time 10 h after culture in monoculture. For QTL S2217304 from *S. aureus*, its maximum effect occurs at 3 and 8 h in co-culture and monoculture, respectively. Furthermore, this QTL changes the direction of its genetic effect from monoculture to co-culture.

### Mapping the Two-Dimensional Genetic Architecture of Microbial Growth in Co-culture

**FIGURE 4:**
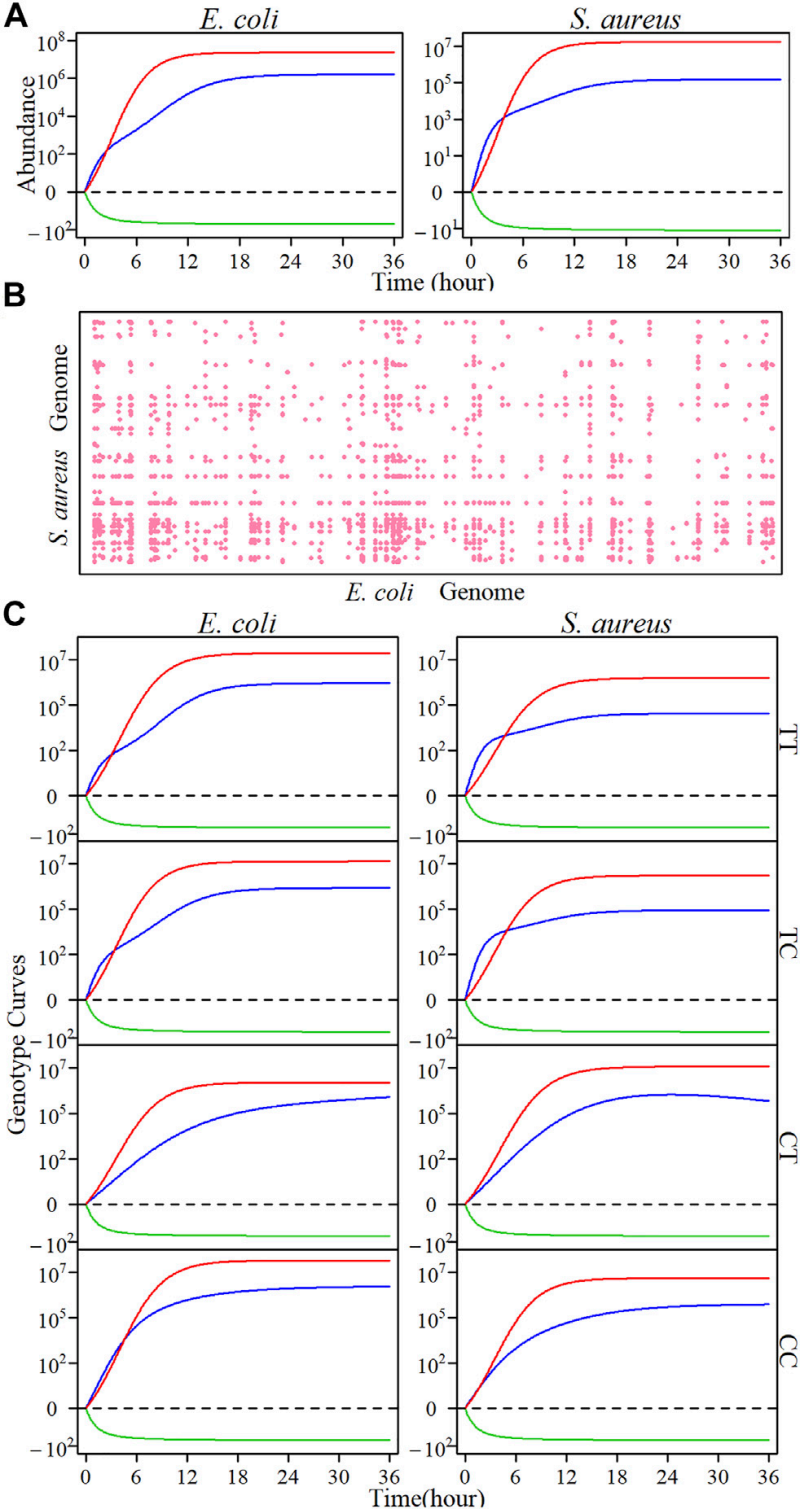
The two-dimensional genetic analysis results of interspecies interactions between *E. coli* and *S. aureus* in community growth. **(A)** The overall microbial abundance (blue line) of *E. coli* and *S. aureus* in co-culture, and the underlying independent (red line), and interactive (green line) growth components. **(B)** Two-dimensional Manhattan plots of significant QTL pairs on two microbial genomes. The genome-wide significance threshold is determined by $10^{-6}$ significance level after Bonferroni correction. **(C)** Microbial growth curves of four genotype combinations T/T, T/C, C/T, and C/C formed by QTL E4320079 from *E. coli* and S188004 from *S. aureus*.

Unlike a case of monoculture in which the phenotype of a species is only controlled by its own genes, the growth of one species may be controlled epistatically by its own genome and the genome of its co-existing species in co-culture. We find that in co-culture, *E. coli* and *S. aureus*, tend to be antagonistic; i.e., one species grows at a cost of the other species, with the strength of such antagonism being quantified by the HollinLV model Eq. 1 (Figure 4A). Our mapping model can characterize significant interspecifically epistatic SNP pairs that affect a species’ growth trajectory in co-culture. By pairwise scanning loci, each from a different species, throughout the *E. coli* and *S. aureus* genomes, we plot a two-dimensional Manhattan plot that demonstrates 2,245 significant interspecific SNP pairs comprising of 249 SNPs from *E. coli* and 182 SNPs from *S. aureus* (Figure 4B). We determined the critical threshold at the significance level of 10^−6^ after Bonferroni correction. The functional annotation of genes from NCBI’s database shows that almost all QTLs detected residue within candidate genes of known biological functions. For example, for *E. coli*, E19056 resides in gene *nhaR* activating the distal promoter, *osmCp1*, of expression of *osmC*. It can stimulate osmCp1 in response to an osmotic signal (Sturny et al., 2003; Katarzyna et al., 2010). E49080 resides in gene *kefC*, which is a potassium transport system regulated by glutathione metabolites (Miller et al., 2000). E62059 is relevant to the *rapA* controlling an additional mechanism, which involved in wild-type biofilm resistance (Lynch et al., 2007). For *S. aureus*, S188004 exerts pronounced genetic interactions with many SNPs distributed over the *E. coli* genome, it resides in gene *ggt* associating with the gammaglutamyltranspeptidase facilitating glutathione utilization (Hussain and Roslinah, 2008).

At each SNP pair, there are four possible genotype combinations between two species. To show how a SNP from different genomes interacts with each other to affect microbial abundance, we decompose the net growth curves of each genotype combination into independent curves and dependent curves for each species. Figure 4C illustrates such an example from SNPs E4320079 with two genotypes T and C residing within the region of *phnI* gene and S188004 with two genotypes T and C residing within the region of *ggt* gene, producing combinations TT, TC, CT, and CC. We find that net, independent, and dependent growth curves for each species vary among four genotype combinations; for example, CT displays less independent growth in *E. coli* than the other combinations, whereas there is less independent growth in *S. aureus* for TT than the other combinations. For all combinations, *E. coli* and *S. aureus* are antagonistic, but with the strength of antagonism depending on genotype combination, suggesting that SNP pair E4320079 and S188004 plays a role in mediating the microbial growth of two coexisting species.

**FIGURE 5:**
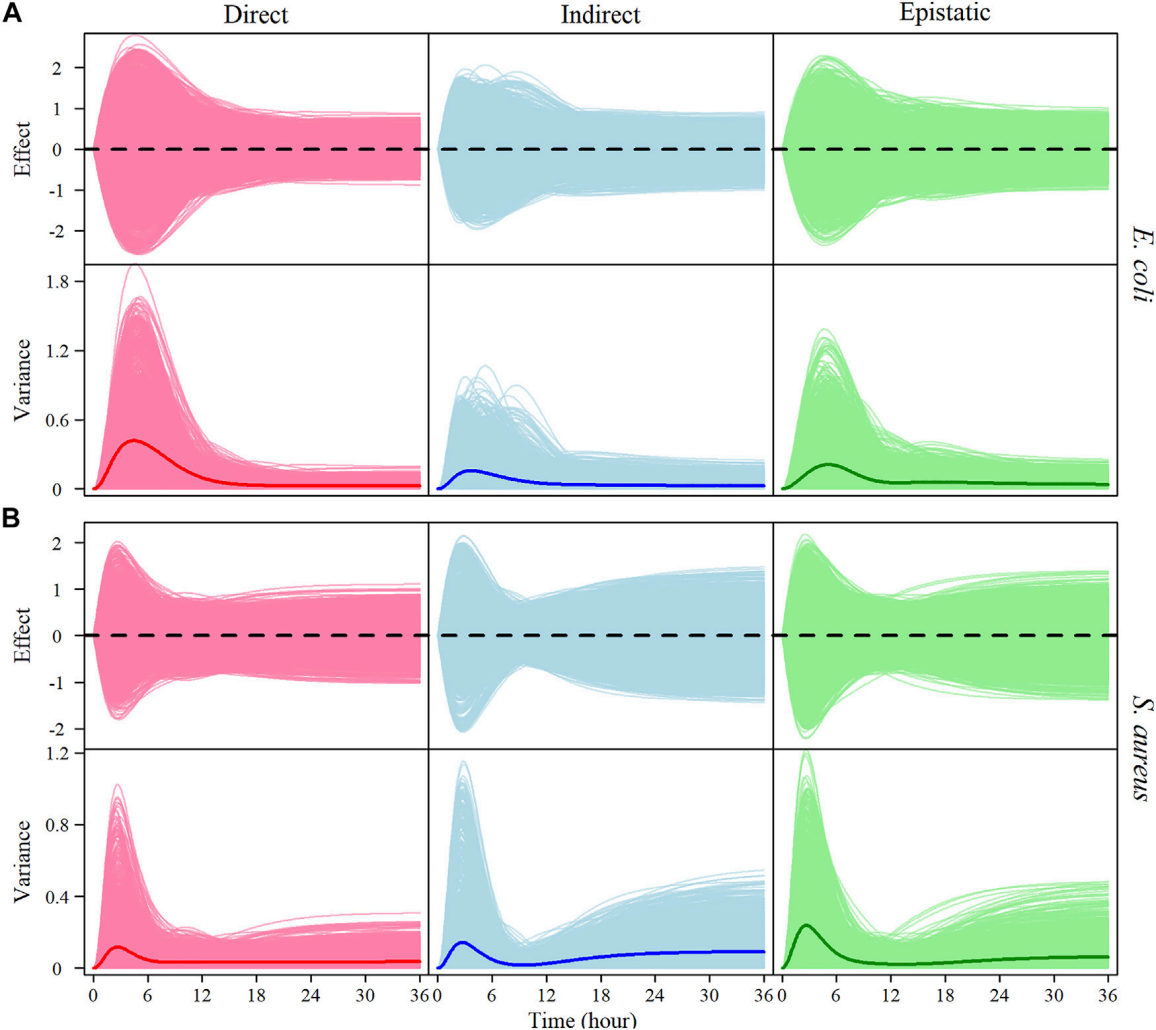
The genotypic value of each genotype combination is partitioned into its direct (red), indirect (blue), and genome–genome (G–G) epistatic effects (green) on the growth, with the time-varying genetic variance explained by each of these effects of *E. coli* **(A)** and *S. aureus* **(B)** in co-culture. The thick lines on the variance curves represent the average variance of direct, indirect, and epistatic effects, respectively.

We implemented Jiang et al. (2018) model to partition the genotypic values of each significant interspecific SNP pair into direct genetic effects, indirect genetic effects, and genome-genome epistatic effects on the growth trajectory of each species. Based on the estimates of these effect values, we calculate the genetic variance curves due to each effect. It is interesting to see that the growth of each species in co-culture is not only affected directly by its own genes, but also, to a similar extent, indirectly by genes from its co-existing conspecific and epistatically by cross-genome interactions of the gene between two species (Figure 5).

### Computer Simulation

To investigate the utility and the statistical properties of HollinLV, we performed simulation studies by mimicking the real example as described above. The data were simulated by assuming that two species were reared in monoculture and co-culture. The phenotype was determined by a set of QTLs among 1,000 simulated markers, plus a residual error following a bivariate normal distribution. We simulated *n* = 45, 100, and 200 interspecific pairs in co-culture under heritabilities of H^2^fn2 = 0, 0.05, and 0.10. The size of heritability was used to adjust the magnitude of innovative variance.

Four genotypic curves at a joint locus of two genomes can be reasonably well estimated even using a small sample size under a modest heritability level (H^2^ = 0.05) (Supplementary Figure S3A; Supplementary Table S1). As expected, the accuracy and precision of curve estimation can be increased by increasing sample size and heritability. In general, the level at which estimation precision improves from H^2^ = 0.05 to 0.10 under *n* = 0.45 is similar to the level of improvement from *n* = 45 to 100 under H2 = 0.05 (Supplementary Figure S3B; Supplementary Table S1). Because Jang et al. (2018) experiment was well controlled in a uniform environment, producing minimum noises, we can expect that abundance data contains so adequately large a heritability that small sample size *n* = 45 can provide reasonably precise estimation of genotypic curves by our HollinLV model.

We performed computer studies to investigate the empirical power of HollinLV for QTL detection under different samples and heritabilities (Table 2). When both heritability and sample size are small (H^2^ = 0.05 and *n* = 45), the model’s power is quite low (0.37), but it increases dramatically when H^2^ increases to 0.10 (0.76) or when n increases to 100 (0.85). As discussed above, Jiang et al. (2018) experiment might have increasing heritability because of well-controlled environment. However, the results from such a small sample size should be interpreted with caution. When sample size increases to 200, the power of QTL detection can increase to >0.96 even with a small heritability. Therefore, in practice, a sample size of *n* = 200 is recommended for our HollinLV model to detect interaction QTLs. In general, the model has a low false positive rate, especially when sample size is 100 or higher (Table 2).

**TABLE 2:** The positioning accuracy and false positive probability of significant QTLs identified by DDHR. The experiments simulated the co-culturing 45 pairs (the same as the real example), 100, pairs and 200 pairs under heritability levels of 0, 0.05 and 0.10. The positioning accuracy and false positive probability were evaluated by computer simulation.

Heritability		Size		
		45	100	200
FPR	0	0.11	0.07	0.08
Power	0.05	0.372	0.855	0.957
	0.1	0.76	0.973	0.985

## Discussion

Community genetics has emerged as a subdiscipline of genetics that combines community ecology and ecological genetics to gain insight into the genetic mechanisms underlying phenotypic diversity and evolution within and between species (Whitham et al., 2006; Hersch-Green et al., 2011). In this article, we develop and implement a computational model to address three fundamental questions in community genetics: 1) how a given species genetically adapts to its coexisting conspecifics, 2) which genetic machineries mediate a species’ phenotypes expressed in ecological communities, and 3) what is the genetic architecture underlying interspecific interactions. This model is the integration of systems mapping and community ecology through a system of HollinLV equations. Systems mapping views and maps a complex trait as a system whose interconnected components act together to mediate the trait, following a similar principle of community ecology, i.e., different species interact with each other to shape community dynamics.

There has been a rich body of literature on studying the genetic control of the phenotypic response of an organism to abiotic environment (Wang et al., 2013; Jiang et al., 2018; Diouf et al., 2020), but knowledge about how genes act differently in response to biotic environment is very limited. It is impossible to answer this question without the integration of quantitative genetic models and ecological data. Our HollinLV model has made this integration possible. An ecological experiment generates data from socially isolated environments (monoculture) and socialized environment (co-culture). HollinLV can test the change of genetic control in terms of the number and action of gens involved from monoculture to co-culture, providing insight into how a given species genetically responds to the co-existence of other species. By analyzing a real dataset, HollinLV identifies a set of specific QTLs that are activated by the existence of other species. In the co-culture of two bacterial species *E. coli* and *S. aureus*, a high percentage of QTLs are detected to be different from those expressed in monoculture. Such a percentage is highly species dependent; e.g., 75.76% for *E. coli* and 91.30% for *S. aureus*. Many more new QTLs activated for *S. aureus* in response to *E. coli* than for *E. coli* in response to *S. aureus* may be due to a higher degree of inhibition for *S. aureus* by *E. coli* than for *E. coli* by *S. aureus*. These discoveries could have immediate implications; for example, if altering the expression of genes of *S. aureus* that are related to with interactions with *E. coli*, the abundance of *S. aureus* can be controlled. A similar genetic manipulation can be made to control the abundance of *E. coli*.

Classic quantitative genetic theory suggests that the phenotype of an organism is controlled by its genes and surrounding environment (Lynch and Walsh, 1997). However, this theory does not interpret how a phenotypic trait is genetically controlled in ecological communities in which intra- and interspecific interactions are also a driver of phenotypic variation (Whitham et al., 2006; Bailey et al., 2009; Hersch-Green et al., 2011; Miner et al., 2012; Crutsinger, 2016; Wimp et al., 2019). By integrating community ecology theory, our HollinLV model equips quantitative genetic theory with new power to more comprehensively decipher the genetic landscape of biological diversity and community behavior. HollinLV models the phenotypic variation of a species through a joint genetic action of different genomes each from a co-existing species. This modeling framework allows us to dissect genetic control into direct genetic effects (due to genes from a given species’ own genome), indirect genetic effects resulting from genes from the genomes of other species coexisting with this species, and genome-genome epistatic effects (arising from the interaction between genes from co-existing species). Classic quantitative genetic approaches can only estimate the direct genetic effects of a complex trait, whereas our HollinLV model leverages these approaches to characterize the impacts of indirect and trans-genome interaction effects. By analyzing Jiang et al. (2018) real data, we find that the phenotype of a bacterial species is determined not only by its own genes, but also by the genes of its coexisting conspecific and, remarkably, by the interaction of genes from two species. In general, *E. coli*’s genes are found to exert more impactful indirect effects on the abundance of *S. aureus* than those of *S. aureus*’ genes on *E. coli* abundance (Figure 5), showing that *E. coli* plays a dominant role in shaping the coexistence of two species. Furthermore, trans-genome interactions are an important component of the genetic architecture underlying each species’ abundance. The precise dissection of overall genetic control over community phenotypes by HollinLV can greatly enhance our understanding of community behavior, dynamics and evolution.

HollinLV divides the net (observed) phenotype of a species into two components, the independent component arising from this species’ intrinsic capacity and the dependent components due to the influence of other species on it, and quantifies each of these two components. The sign and size of the dependent components for a pair of species characterize all possible patterns of ecological interactions from mutualism to commensalism to amensalism to predation to antagonism (Diouf et al., 2020). HollinLV allows us to characterize the genetic control mechanisms of each type of interaction and compare how the same genes differently govern these interactions. In Jiang et al. (2018) data, two bacterial species were found to be antagonistic to each other, although the strength of antagonism is larger for *E. coli* towards *S. aureus* than for *S. aureus* towards *E. coli*. Specific QTLs from each species have been identified to mediate their antagonism in co-culture. With these antagonism-related QTLs and the corresponding genes, microbial geneticists can destroy this antagonistic relationship or change it to other types of interactions with aid of gene editing techniques.

HollinLV is an ecology-oriented mapping model that can potentially gain new insight into community genetics. Its application to a microbial cultural experiment shows some promise to better understand the genetic control of interspecific interactions between two bacterial species. However, results from our analysis should be interpreted with caution because the data contains a small sample size. The future study should increase sample size to 200 interspecific pairs by which parameter estimation and detection power can reach a satisfactory level. Also, the model derivation is based on a pair of species in co-culture. This obviously is not adequate to be applied to practical ecological communities where many species are coexisting and interact with each other to form a complex ecological network. Holling-typed response models only characterize an aspect of community systems, and more comprehensive models are required to better capture multifaced features of communities. Regardless, our HollinLV provides a start point for integrating quantitative genetic theory and community ecology to disentangle the complexity of community genetics.

## Data Availability

Publicly available datasets were analyzed in this study. This data can be found here: The raw sequence data were deposited in the NCBI short reads archive under accession number SRP074089 and SRP074912.
