# Supplementary material for: A Holling Functional Response Model for Mapping QTLs Governing Interspecific Interactions
Source: Front Genet. 2021 Oct 15;12:766372. doi: 10.3389/fgene.2021.766372 (PMC8554200; doi:10.3389/fgene.2021.766372)
Supplement: Supplementary file 1 [file DataSheet1.PDF]

Supplementary Material

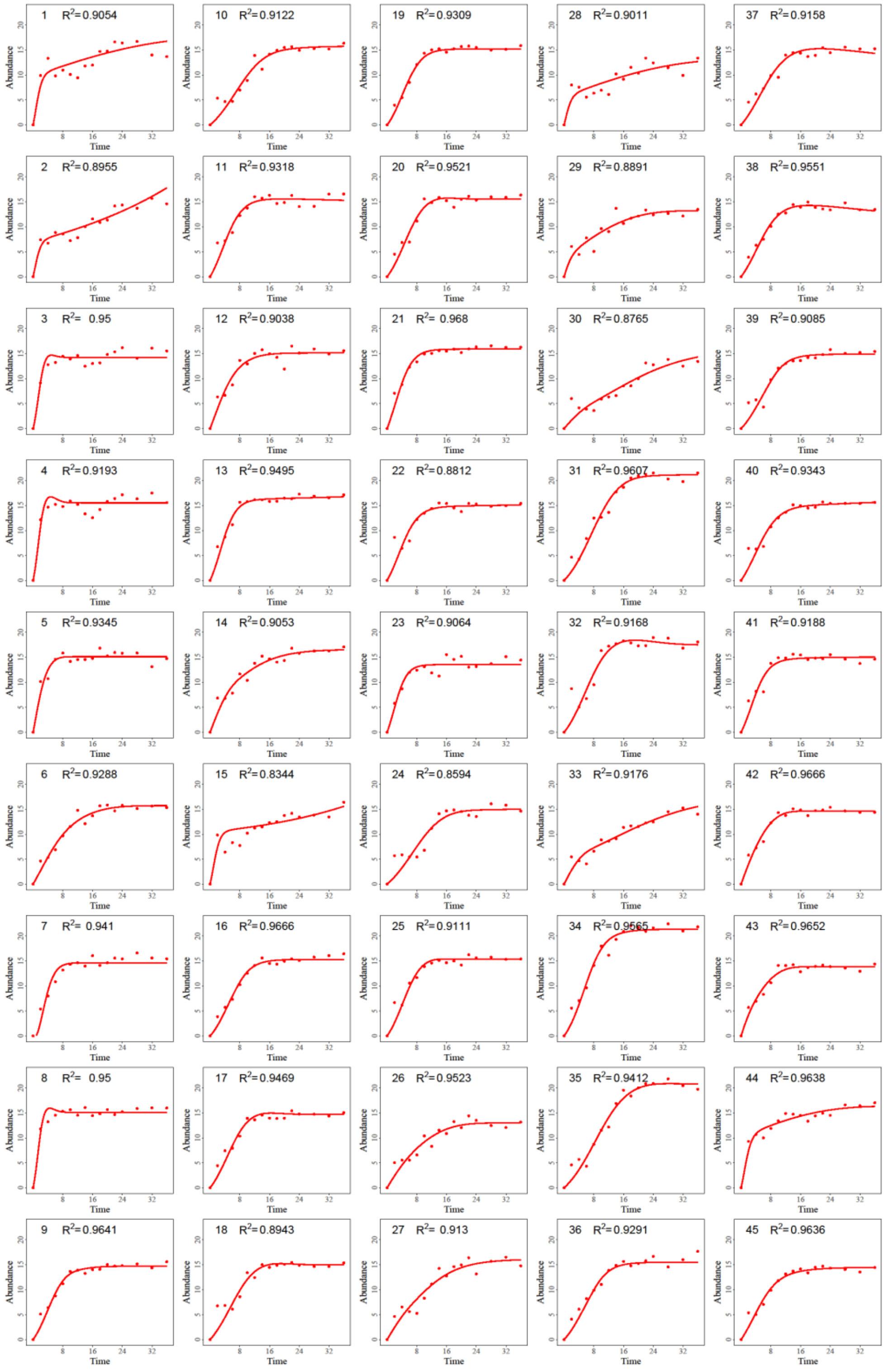

Supplementary Figure S1. Growth curves of 45 E. coli individual strains in co-culture. The observed microbial abundance was shown as points. Goodness-of-fit (R^2) of HollinLV for each stain was calculated.

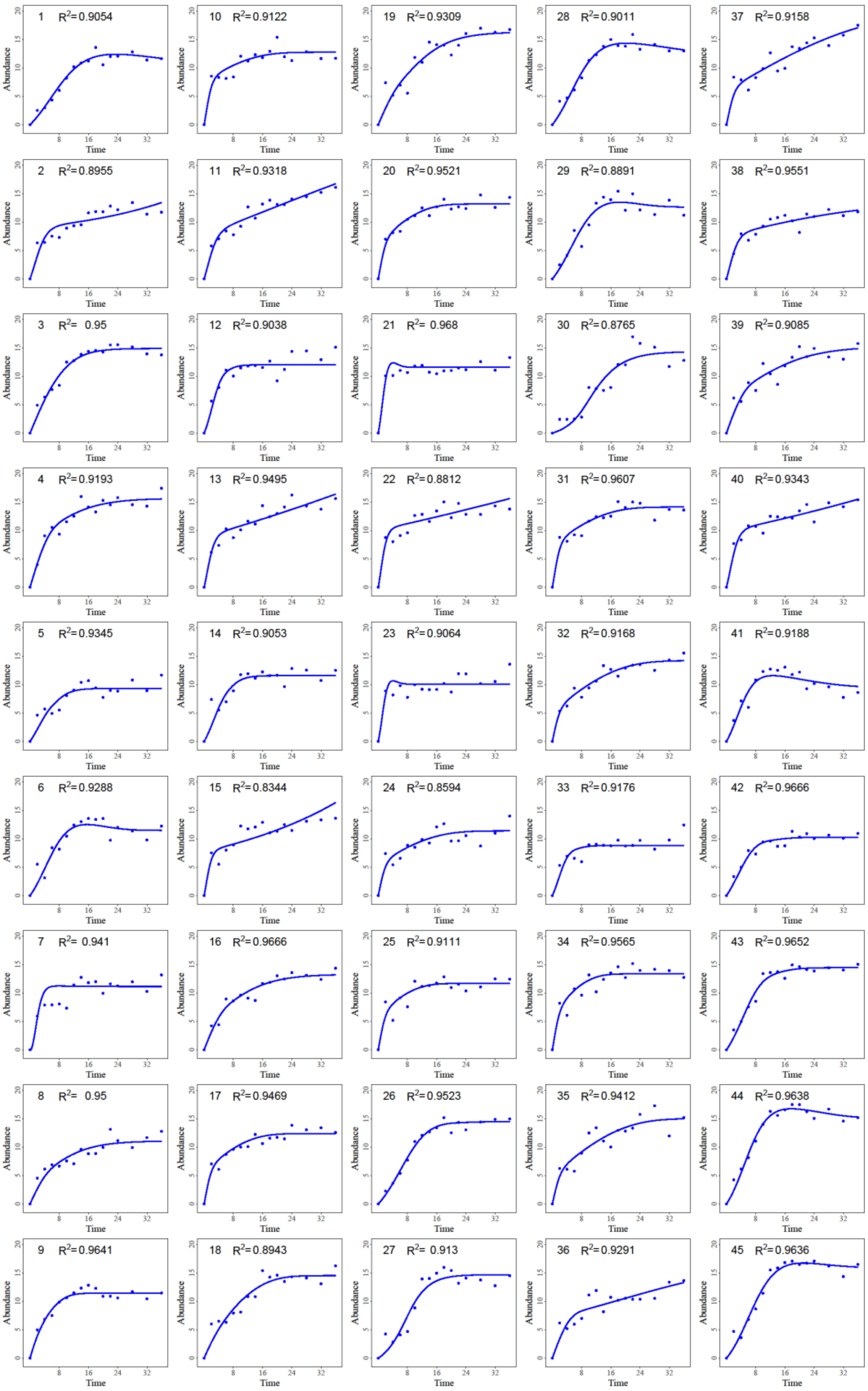

Supplementary Figure S2. Growth curves of 45 *S. aureus* individual strains in co-culture. The observed microbial abundance was shown as points. Goodness-of-fit (R^2) of HollinLV for each stain was calculated.

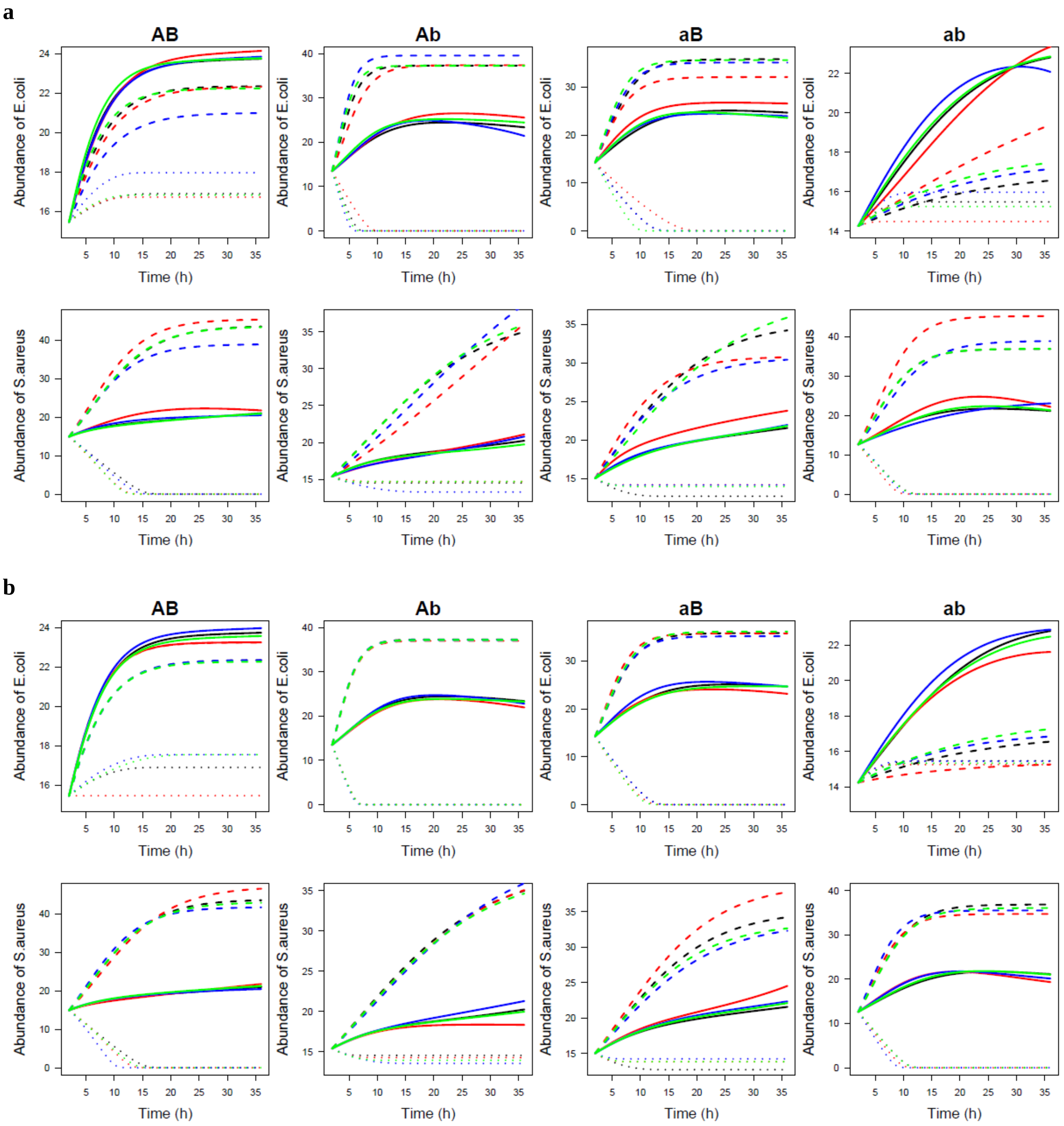

Supplementary Figure S3. Growth curves of four genotype combinations across different species in co-culture under the heritability of 0.05 (**a**) and 0.10 (**b**). The overall growth of microbial abundance (solid line) for each combination is decomposed into its independent (broke line) and dependent growth components (dot line) for *E. coli* and *S. aureus*. The sample size is 45,100 and 200, respectively. Black line is the real curve and red line, blue line, and green line are the estimated curves under the sample size of 45, 100, and 200, respectively.

Supplementary Table S1. Estimation of HollinLV parameters and their standard deviations (in parentheses) at different genotype combinations AB. Ab, aB, and ab under different samples of n = 45, 100, and 200 and different heritabilities (H2 = 0.05, 0.10).

			r_e	K_e	$\alpha_{E\leftarrow S}$	r_s	K_s	$\alpha_{S\leftarrow E}$
True		AB	0.2103	22.3519	0.0748	0.1770	43.6858	-0.4548
		Ab	0.5224	37.3031	-0.4629	0.0867	37.6294	-0.3633
		aB	0.3177	35.8507	-0.3810	0.1131	35.2283	-0.3179
		ab	0.0556	17.0489	0.4282	0.2644	36.8571	-0.4288
$H^2 = 0.05$	n=45	AB	0.1334<br>(0.1198)	11.9735<br>(7.2280)	3.4253<br>(4.7584)	0.1579<br>(0.1228)	48.0151<br>(18.3740)	-0.4096<br>(0.1684)
		Ab	0.5598<br>(0.2893)	38.9335<br>(6.5144)	-0.4637<br>(0.1088)	0.1219<br>(0.0921)	46.0338<br>(21.3441)	-0.3414<br>(0.1944)
		aB	0.5921<br>(0.2351)	41.2133<br>(6.8986)	-0.4716<br>(0.1267)	0.1017<br>(0.0840)	46.7111<br>(24.1166)	-0.4649<br>(0.3776)
		ab	0.0393<br>(0.1179)	20.1734<br>(19.5490)	3.2992<br>(3.8910)	0.4355<br>(0.3219)	44.2830<br>(16.9875)	-0.4473<br>(0.2031)
	n=100	AB	0.1903<br>（0.1391）	14.3989<br>（6.5622）	1.6497<br>（2.2091）	0.2070<br>（0.0816）	48.6720<br>（21.2146）	-0.4430<br>（0.1343）
		Ab	0.4650<br>（0.1905）	34.4017<br>(5.6031)	-0.3671<br>(0.1395)	0.1240<br>(0.1682)	42.2961<br>(12.3364)	-0.3556<br>(0.1439)
		aB	0.5128<br>(0.1957)	38.3552<br>(5.1759)	-0.4407<br>(0.1248)	0.2508<br>(0.5556)	41.6526<br>(13.0393)	-0.3478<br>(0.1233)
		ab	0.0912<br>(0.1689)	11.3283<br>(6.4163)	2.3294<br>(2.3569)	0.4488<br>(0.3612)	41.8477<br>(10.4678)	-0.4395<br>(0.1588)
	n=200	AB	0.1511<br>(0.0675)	15.1597<br>(5.8739)	1.3172<br>(1.9208)	0.1750<br>(0.0622)	45.3798<br>(12.1830)	-0.4321<br>(0.1341)
		Ab	0.4711<br>(0.1448)	36.2635<br>(4.3333)	-0.4177<br>(0.1246)	0.0884<br>(0.0442)	40.5441<br>(6.9766)	-0.6998<br>(1.6728)
		aB	0.3920<br>(0.1378)	37.3351<br>(6.3679)	-0.3981<br>(0.1519)	0.1257<br>(0.0873)	41.3683<br>(10.7192)	-0.3556<br>(0.1140)
		ab	0.0496<br>(0.0378)	15.7787<br>(9.1073)	1.3389<br>(1.4028)	0.2867<br>(0.0906)	38.8906<br>(9.5649)	-0.4268<br>(0.1473)
$H^2 = 0.1$	n=45	AB	0.1294<br>(0.0767)	12.7163<br>(6.7160)	2.5713<br>(3.3689)	0.2039<br>(0.1141)	52.3194<br>(15.7459)	-0.4725<br>(0.1247)
		Ab	0.5327<br>(0.1703)	35.9761<br>(5.1503)	-0.4167<br>(0.1371)	0.0739<br>(0.0769)	41.7029<br>(14.8102)	-0.9032<br>(1.0685)
		aB	0.5253<br>(0.3759)	38.4927<br>(8.6440)	-0.4046<br>(0.1922)	0.1476<br>(0.0964)	45.0169<br>(18.6892)	-0.8749<br>(2.1947)
		ab	0.0754<br>(0.1564)	16.9576<br>(11.3751)	1.8415<br>(2.0836)	0.5008<br>(0.5397)	42.4071<br>(13.0569)	-0.430<br>(0.1890)
	n=100	AB	0.1576<br>(0.0686)	16.0891<br>(5.7289)	1.0336<br>(1.3852)	0.1804<br>(0.0573)	46.0843<br>(13.1310)	-0.4362<br>(0.1360)

		Ab	0.4903<br>(0.1410)	36.2509<br>(4.5683)	-0.4222<br>(0.1161)	0.0822<br>(0.0443)	40.3231<br>(13.8735)	-0.4790<br>(0.5760)
		aB	0.3817<br>(0.1313)	37.6797<br>(6.9400)	-0.4006<br>(0.1611)	0.1151<br>(0.0660)	39.9032<br>(10.6261)	-0.3625<br>(0.2981)
		ab	0.0385<br>(0.0305)	14.0393<br>(9.2380)	1.9532<br>(2.1711)	0.2945<br>(0.0843)	38.9329<br>(9.3966)	-0.4265<br>(0.1609)
	Size=200	AB	0.1586<br>(0.0686)	16.6754<br>(5.7289)	0.8118<br>(1.3852)	0.1644<br>(0.0573)	42.0366<br>(13.1310)	-0.4009<br>(0.1360)
		Ab	0.4963<br>(0.1410)	36.2680<br>(4.5683)	-0.4261<br>(0.1161)	0.0867<br>(0.0443)	37.0767<br>(13.8735)	-0.3310<br>(0.5760)
		aB	0.3405<br>(0.1313)	37.0554<br>(6.9400)	-0.3877<br>(0.1611)	0.1187<br>(0.0660)	38.7329<br>(10.6261)	-0.3164<br>(0.2981)
		ab	0.0398<br>(0.0305)	15.6362<br>(9.2380)	1.2132<br>(2.1711)	0.2830<br>(0.0843)	37.1539<br>(9.3966)	-0.4030<br>(0.1609)
